# Supplementary material for: Murine Gammaherpesvirus 68 ORF45 Stimulates B2 Retrotransposon and Pre-tRNA Activation in a Manner Dependent on Mitogen-Activated Protein Kinase (MAPK) Signaling
Source: Microbiol Spectr. 2023 Feb 8;11(2):e00172-23. doi: 10.1128/spectrum.00172-23 (PMC10100704; doi:10.1128/spectrum.00172-23)
Supplement: Supplemental file 1 — Supplemental material. Download spectrum.00172-23-s0001.pdf, PDF file, 0.2 MB [file spectrum.00172-23-s0001.pdf]

Supplemental Figure 1

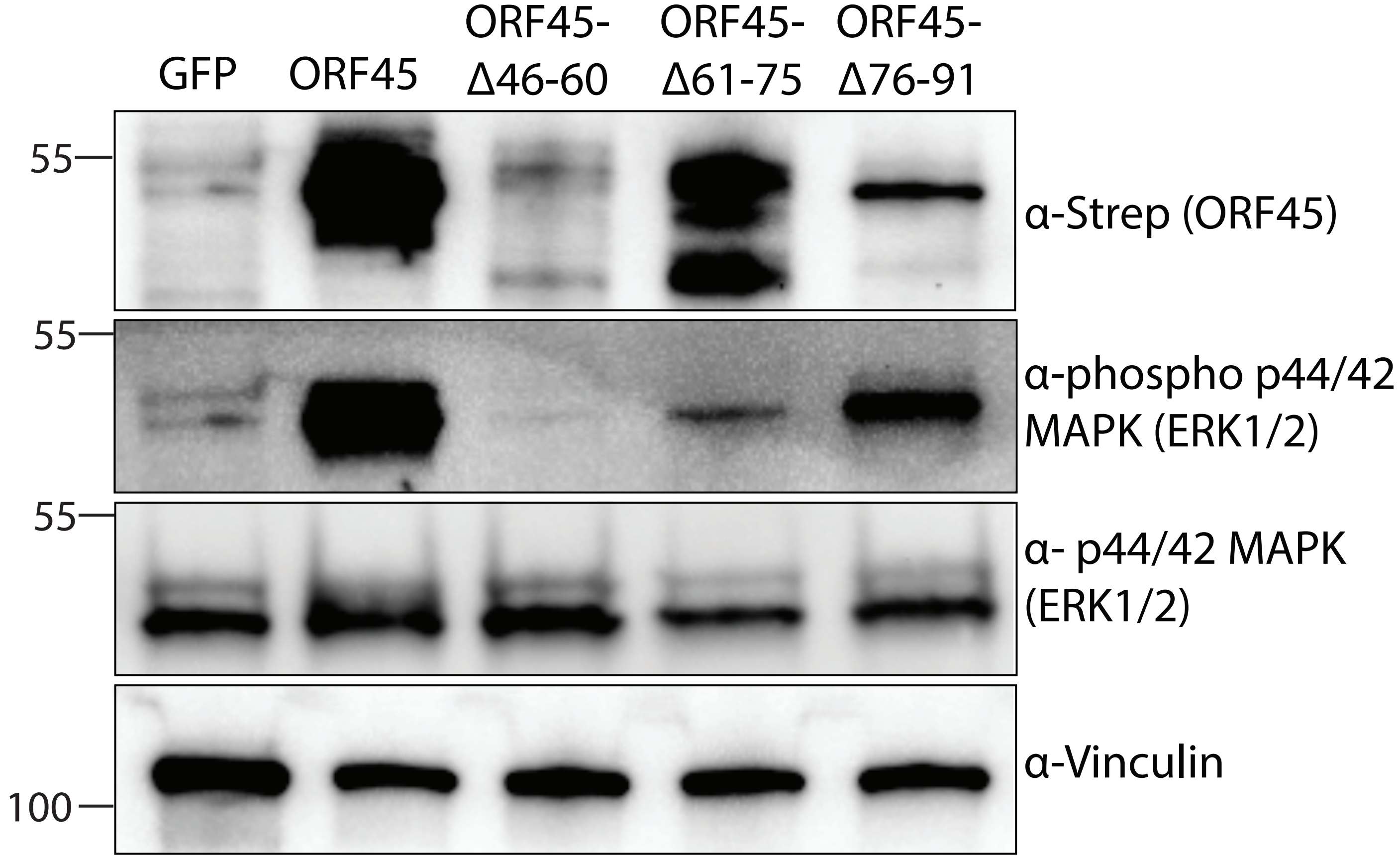

SUPPLEMENTAL FIG 1 ORF45 deletion mutants show differential activation of ERK. NIH 3T3 fibroblasts were transfected with indicated Strep-tagged ORF45 genes or a GFP control for 24h, whereupon lysates were analyzed by Western blotting with antibodies against Strep, phospho-ERK1/2, ERK, and Vinculin (loading control).

Supplemental Table 1: FLAG-tagged MHV68 ORFs screened for a role in B2 SINE transcriptional activation

co-tansfection group:	proposed functions:	ORF name:	B2 SINE fold activation compared to GFP control (measured by primer extension)	individiual ORF tranfection repeated in triplicate (>2 fold cutoff)?	verfied as reproduciple inducer of B2 SINE transcription (>2 fold in 3 replicates)?
		K3		x	
1	cell signaling	M11	1.5	x	
		M2		x	
		M4		x	
2	unknown	M8	1.6	x	
		ORF6		x	
3	DNA synthesis	ORF9	1.1	x	
		ORF56		x	
4	DNA synthesis	ORF59	1.4	x	
		ORF17		x	
5	capsid	ORF25	0.8	x	
		ORF18b		x	
6	unknown/nuclear egress	ORF69	0.8	x	
		ORF19		x	
7	tegument/glycoprotein	ORF39	1.3	x	
		ORF21		x	
8	DNA synthesis	ORF54	1.4	x	
	DNA	ORF62		x	
9	maturation/transcription	ORF73	1.3	x	
		ORF64		x	
10	tegument	ORF75a	0.9	x	
		ORF75b		**yes**	no
11	tegument	ORF75c	3.5	**yes**	no
		ORF65		**yes**	no
12	capsid/tegument	ORF45	2.4	**yes**	**yes**
